# Deciphering the lactylation landscape in glioma: a novel gene signature predicts patient survival and immunotherapy sensitivity

**DOI:** 10.3389/fimmu.2025.1664347

**Published:** 2025-09-16

**Authors:** Xiaolong Tang, Yi Liu, Congying Yang, Honglan Zhang, Gongming Zhang, Qiao Wang, Sujie Jiang, Xuzhu Gao, Yongshuo Liu, Yanbin Dong

**Affiliations:** ^1^ Department of Laboratory Medicine, Hospital of Chengdu University of Traditional Chinese Medicine, Chengdu, Sichuan, China; ^2^ Department of Pathology, The Affiliated Lianyungang Hospital of Xuzhou Medical University, Lianyungang Clinical College of Nanjing Medical University, The First People’s Hospital of Lianyungang, Lianyungang, China; ^3^ Department of Information, The Affiliated Lianyungang Hospital of Xuzhou Medical University, The First People’s Hospital of Lianyungang, Lianyungang, China; ^4^ Department of Central Laboratory, The Second People’s Hospital of Lianyungang, Cancer Hospital of Lianyungang, Lianyungang, China; ^5^ Department of Clinical Laboratory, Shandong Cancer Hospital and Institute, Shandong First Medical University and Shandong Academy of Medical Sciences, Jinan, China

**Keywords:** glioma, lactylation, HSPE1, prognosis, immunotherapy

## Abstract

**Background:**

Glioma, the most prevalent primary brain tumor, takes advantage of lactylation, a metabolic modification linked to tumor behavior and clinical outcomes. Despite its significance, the role of lactylation in the pathogenesis and prognosis of glioma remains underexplored. This study established a lactylation-derived molecular signature to predict survival and response to immunotherapy in glioma.

**Methods:**

Leveraging the TCGA glioma cohort, we established a lactylation-related gene (LRG) signature *via* LASSO and Cox regression analyses, and its prognostic value was validated in independent cohorts. We comprehensively characterized the associations between the LRGs signature and clinicopathological features, tumor immunity (immune infiltration and response to immunotherapy), genomic instability (mutational burden and heterogeneity), tumor stemness, and therapeutic vulnerability. *In vitro* validation of the oncogenicity of HSPE1 was conducted using the CCK-8, colony formation, transwell, and apoptosis assays in U87 and U251 glioma cells.

**Results:**

A four-gene lactylation signature (KIF2C, CALD1, HSPE1, and IFI16) was identified. Elevated LRGs score were correlated with advanced tumor grade, poor prognosis, and reduced response to immunotherapy. Patients in the LRGs-high group exhibited adverse clinicopathological features, including advanced age, higher WHO grade, IDH wild-type status, and 1p/19q non-codeletion. The nomogram model based on the LRGs score exhibited robust prognostic accuracy (C-index = 0.860). LRGs-related genes were enriched in immune regulatory pathways, such as cytokine signaling and interferon-γ response pathways. The LRGs-high group displayed increased infiltration of immunosuppressive cells, such as M2 macrophages, MDSCs, and CAFs, and distinct genomic instability profiles. Crucially, HSPE1 knockdown significantly suppressed the proliferation and invasion of glioma cell lines.

**Conclusions:**

We defined a novel LRGs signature integrating metabolic and immune dysregulation in glioma. This signature served as an independent predictor of prognosis and immunotherapy. Furthermore, we identified HSPE1 as a critical driver of glioma progression.

## Introduction

Glioma, the most prevalent primary malignant tumor of the central nervous system, accounts for approximately 81% of adult CNS malignancies. These tumors originate from glial or glial precursor cells and encompass histopathological subtypes including astrocytoma, oligodendroglioma, ependymoma, and oligoastrocytoma (1). The World Health Organization (WHO) classifies malignant gliomas into low-grade glioma (LGG, WHO II and III) and glioblastoma (GBM, WHO IV), with the latter being the most aggressive and lethal subtype (2, 3). Despite multimodal therapy—including maximal safe resection, radiotherapy, alkylating chemotherapy, and emerging immunotherapies—clinical outcomes remain dismal. Specifically, patients with LGG exhibit a median survival of 5–10 years (4), while patients with GBM rarely survive more than 14.6 months (5). Sonodynamic therapy (SDT) is constantly innovating and can potentially be a non-invasive treatment for glioma in the future (6). However, prognosis is critically affected by WHO grade, molecular alterations, such as IDH mutation and 1p/19q codeletion, and therapeutic resistance (7), underscoring an urgent need for more effective prognostic biomarkers and therapeutic targets.

Advances in bioinformatics and high-throughput genomic profiling have revolutionized cancer prognostication (8). Molecular prognostic models can help refine risk stratification, identify therapeutic vulnerabilities, and characterize the tumor immune microenvironment (TIME), guiding precision oncology approaches in glioma and other tumors (9, 10). Such models play an indispensable role in predicting response to immunotherapy and the development of personalized therapeutic strategies.

Lactylation—a recently discovered post-translational modification driven by lactate—is a key regulator of cancer metabolism and epigenetics. This modification affects diverse oncogenic processes, including proliferation, invasion, DNA repair, and treatment resistance (11). In GBM, the lactylation of XRCC1 enhanced nuclear translocation and DNA repair capacity, promoting resistance to chemoradiotherapy (12). Despite its established role in tumor biology, the prognostic significance of lactylation-related genes in glioma remains largely unexplored, representing a critical knowledge gap.

To address this gap, we constructed and validated a novel prognostic LRGs signature based on four genes (KIF2C, CALD1, HSPE1, and IFI16). This signature stratified patients into distinct risk groups with significant survival differences and was associated with immunosuppressive TIME features—particularly M2 macrophage infiltration—predicting a weak response to immunotherapy. Further analyses revealed differential genomic instability, tumor stemness, and therapeutic vulnerabilities between LRGs-high and -low groups. Critically, functional validation confirmed HSPE1 as a pro-oncogenic driver, where its silencing suppressed glioma cell proliferation and invasion *in vitro*. Our integrated analysis established the lactylation-derived signature as a robust biomarker for prognosis and treatment response in glioma.

## Materials and methods

### Data source

The glioma and other cancer cohorts used in this study were sourced from The Cancer Genome Atlas (TCGA) (https://portal.gdc.cancer.gov/), Chinese Glioma Genome Atlas (CGGA) (http://www.cgga.org.cn/), Gene Expression Omnibus (GEO) (https://www.ncbi.nlm.nih.gov/geo/), and Kaplan-Meier Plotter (https://kmplot.com/) databases, with detailed information provided in **Table 1**. According to previously published study (13, 14), a total of 354 lactylation-related genes were included and were presented in **Supplementary Table S1**.

**Table 1:** Details of the cohorts used in this study.

Datasets	Normal tissues (N)	Tumor tissues (N)	Clinical data (N)
TCGA	5	701	698
GSE16011	8	276	NA
GSE43378	NA	50	50
CGGA301	NA	285	285
CGGA325	NA	313	313
CGGA693	NA	657	657
GSE4412	NA	85	85
GSE43378	NA	50	50
GSE131928	NA	28	NA
PRJNA482620	NA	34	34
GSE91061	NA	101	101
KM Plotter (Atezolizumab)	NA	447	447

### Construction of a LRGs signature

To construct a lactylation-related gene signature, we first utilized the TCGA glioma cohort to screen for genes associated with patient prognosis, conducting batch survival analysis using the R package “survival”. The resulting prognostic genes were intersected with 354 lactylation-related genes, followed by further refinement of the overlapping genes through LASSO regression analysis using the R package “glmnet”. Univariate and multivariate Cox proportional hazards regression analyses were employed to develop the LRGs model, with Cox regression performed using the R package “survival” and forest plot visualization achieved through the R package “ggplot2”. The chromosomal locations of the four LRGs core genes (KIF2C, CALD1, HSPE1, and IFI16) were visualized using the R package “circlize”, and Spearman’s correlation analysis was employed to examine the relationships among these genes. Glioma patients were stratified into LRGs-high and -low groups using the median LRGs score as a cutoff value. Kaplan-Meier survival analysis and visualization were performed using the R packages “survival” and “survminer” respectively. Scatter plot was generated with the R package “ggplot2”, while time-dependent ROC was produced and visualized using the R packages “timeROC” and “ggplot2”.

### Validation of the LRGs expression levels and prognostic value

To validate the expression patterns of the LRGs, we utilized the TCGA and GSE16011 cohorts to compare the differences of the LRGs score and its core genes between normal brain and glioma tissues. Subsequently, multiple independent cohorts, such as CGGA301, CGGA325, CGGA693, GSE4412, and GSE43378, were employed to evaluate the prognostic value of the LRGs. The methodologies for generating Kaplan-Meier survival curves, scatter plots, and time-dependent ROC curves were consistent with those described previously.

### Clinical relevance of the LRGs and construction of a nomogram model

The TCGA and CGGA693 glioma cohorts were employed to compare differences in gender, age, WHO grade, IDH status, and 1p/19q co-deletion between LRGs-high and -low groups of glioma patients. Based on the TCGA glioma cohort, we incorporated five variables, including age, WHO grade, IDH status, 1p/19q co-deletion, and LRGs score, to construct a predictive nomogram model. Cox regression analysis was performed using the R package “survival”, while the nomogram was developed and calibration curves were plotted using the R package “rms”. Time-dependent AUC and Decision curve analysis (DCA) were analyzed with the R packages “timeROC” and “stdca.R”, respectively, with visualization implemented using the R package “ggplot2”.

### Functional enrichment analysis

Glioma patients in the TCGA cohort were divided into LRGs-high and -low groups according to the median LRGs score as the cutoff value, and differential expression analysis of the original Counts matrix was performed using the R package “DESeq2”. Differentially expressed genes (DEGs) were subjected to enrichment analysis through the Metascape platform (https://metascape.org/). Concurrently, Gene Ontology (GO), Kyoto Encyclopedia of Genes and Genomes (KEGG), and Gene Set Enrichment Analysis (GSEA) were conducted on the DEGs using the R package “clusterProfiler” for analysis and R package “ggplot2” for visualization.

### Tumor microenvironment analysis

The immune cell infiltration scores for glioma patients in the TCGA cohort were obtained from the CIBERSORTx online platform (https://cibersortx.stanford.edu/) based on the Cibersort core algorithm. The infiltration of myeloid-derived suppressor cells (MDSCs) and cancer-associated fibroblasts (CAFs) were derived from the Tumor Immune Dysfunction Exclusion (TIDE) online platform (http://tide.dfci.harvard.edu/). The Stromal, Immune, and ESTIMATE Scores were sourced from the Estimation of STromal and Immune cells in MAlignant Tumor tissues using Expression data (ESTIMATE) database (https://bioinformatics.mdanderson.org/estimate/). We compared the differences in the aforementioned parameters between the LRGs-high and -low groups, and evaluated the correlations between the LRGs core genes and these parameters. The immune subtypes in glioma were sourced from a previously published study (15). Kaplan-Meier survival analysis was performed to assess the significance of immune subtypes in predicting patient survival. The single-cell resolution data from the GSE131928 cohort were sourced from the TISCH2 platform (http://tisch.comp-genomics.org/). We analyzed the differentially expressed genes across various malignant cell types, as well as the expression abundance of LRGs core genes in different cells. Additionally, the biological functions of highly expressed genes in malignant cells were investigated through online analysis using the Metascape platform.

### Immunotherapy predictive capability analysis

The Cancer-immunity cycle was obtained from the Tracking Tumor Immunophenotype (TIP) database (http://biocc.hrbmu.edu.cn/TIP/), which consists of seven steps: (1) release of cancer cell antigens, (2) cancer antigen presentation, (3) priming and activation, (4) trafficking of immune cells to tumors, (5) infiltration of immune cells into tumors, (6) recognition of cancer cells by T cells, and (7) killing of cancer cells. Next, we examined the correlation between the LRGs core genes and the expression of immune checkpoints, such as CTLA4 and PDCD1 etc., using sequencing data from glioma patients in the TCGA cohort. Subsequently, glioma patients were stratified into four groups based on immune checkpoint expression levels combined with LRGs score, and the prognostic significance of these combinations was assessed through Kaplan-Meier survival analysis. Furthermore, we evaluated the predictive capability of the LRGs for immunotherapy efficacy using the TIDE algorithm and multiple cohorts, including glioma (PRJNA482620), melanoma (GSE91061), and atezolizumab pan-cancer (KM Plotter) datasets.

### Genetic mutation analysis

Integrated mutation data for glioma samples were obtained from the GDC portal (https://portal.gdc.cancer.gov/) and analyzed using the R package “maftools” to identify the top 15 most frequently mutated genes between LRGs-high and -low groups. Subsequently, we compared overall survival differences between patients with mutant- and wild-type variants of genes, such as TTN and EGFR etc., and analyzed expression differences of the LRGs core genes between mutant- and wild-type groups.

### Genomic heterogeneity and tumor stemness analyses

Tumor mutation burden (TMB) data were calculated for TCGA glioma samples using the R package “maftools”. Microsatellite instability (MSI) data were referenced from the study by Russell Bonneville et al. (16). Data on tumor purity, tumor ploidy, homologous recombination deficiency (HRD), and neoantigens were obtained from the research by Vesteinn Thorsson et al. (15). Tumor stemness data were derived from previous studies (17), we acquired six stemness indicators based on mRNA expression and methylation signatures, including RNAss, EREG-METHss, DMPss, ENHss, and EREG-EXPss.

### Chemotherapy and radiotherapy sensitivity analyses

For chemotherapy sensitivity analysis, we extracted expression matrix data of glioma patients from the TCGA cohort and utilized the core algorithms of the R packages “oncoPredict” and “pRRophetic” to calculate drug sensitivity scores by integrating drug and cell line expression profiles provided by these tools. For radiotherapy sensitivity analysis, we extracted data from the TCGA glioma patients who received radiotherapy, including those with progressive disease/stable disease (PD/SD) and partial response/complete response (PR/CR).

### siRNA transfection, RNA isolation, and qPCR

siRNAs were purchased from Sangon Biotech. The sequences of the siRNAs were as follows: siCtrl: 5’-UUCUCCGAACGUGUCACGUTT-3’; siHSPE1-1#:5’-GCAGGACAAGCGUUUAGAATT-3’; siHSPE1-2#:5’-GAGUGCUGCUGAAACUGUATT-3’. Glioma cells were seeded in 12-well plates and transfection was initiated when the cell density reached approximately 70%. Transfection was performed using Lipofectamine 3000 according to the manufacturer’s instructions. Transfection efficiency was evaluated by qRT-PCR. Total RNA was extracted using Trizol reagent, and reverse transcription was conducted using M-MLV Reverse Transcriptase. Quantitative real-time PCR was carried out in triplicate using the SYBR Green Master Mix. The following primers were used for qPCR: GAPDH-F: 5’-GTCTCCTCTGACTTCAACAGCG-3’, GAPDH-R: 5’-ACCACCCTGTTGCTGTAGCCAA-3’; HSPE1-F: 5’-GCTGAAACTGTAACCAAAGGAGG-3’, HSPE1-R: 5’- TCTCCAACTTTCACGCTAACTGG-3’.

### Western blot analysis

Western blot analysis was performed as previously described (18). Briefly, glioma cells were lysed using RIPA lysis buffer supplemented with a 1× protease inhibitor cocktail. Equal amounts of protein were separated by 12% SDS-PAGE and transferred to a 0.22 μm PVDF membrane. The membranes were blocked with 5% skimmed milk and then incubated overnight at 4°C with primary antibodies: HSPE1 (Affinity, AF0183) and β-actin (Abmart, P30002). Following primary antibody incubation, the membranes were incubated with the secondary antibody at room temperature for 1 hour. Protein bands were visualized using the Super ECL detection reagent.

### Cell proliferation, colony formation and invasion assays

Cell proliferation was assessed using the Cell Counting Kit-8 (CCK-8), following the manufacturer’s protocol. Absorbance at 450 nm was measured at the indicated time points to determine cell viability. For colony formation assays, 1×10³ cells were seeded in 6-well plates and incubated for 14 days. After incubation, cells were fixed with 4% paraformaldehyde and stained with 0.1% crystal violet to visualize the colonies. For the invasion assay, 5×10^4^ cells were seeded into Transwell inserts with an 8 μm pore size, precoated with Matrigel. After 24 hours of incubation, the invasive cells were stained with 0.1% crystal violet, observed under a microscope, and the number of invasive cells was counted using ImageJ software.

### Cell apoptosis assay

Apoptosis was analyzed using Annexin V-FITC/propidium iodide (PI) double staining followed by flow cytometry. Briefly, harvested cells were resuspended in binding buffer and incubated with Annexin V-FITC and PI for 10 min at room temperature in the dark. The apoptotic cell population was quantified by flow cytometer, and data were analyzed using FlowJo software.

### Statistical analysis

All statistical analyses were processed on R Studio (V4.2.1) or GraphPad Prism 8 software, and *P* value < 0.05 indicated statistically significant differences. The quantitative results are presented as the mean ± standard deviation (SD). *Wilcoxon rank sum* test was used for unpaired samples, *t*-test was used for paired samples, and *ANOVA* was used for comparisons between multiple groups. *Log Rank P* test was used for Kaplan-Meier survival analysis. *Spearman* test was used for Correlation analysis.

## Results

### Development of a LRGs signature for glioma

**Figure 1:**
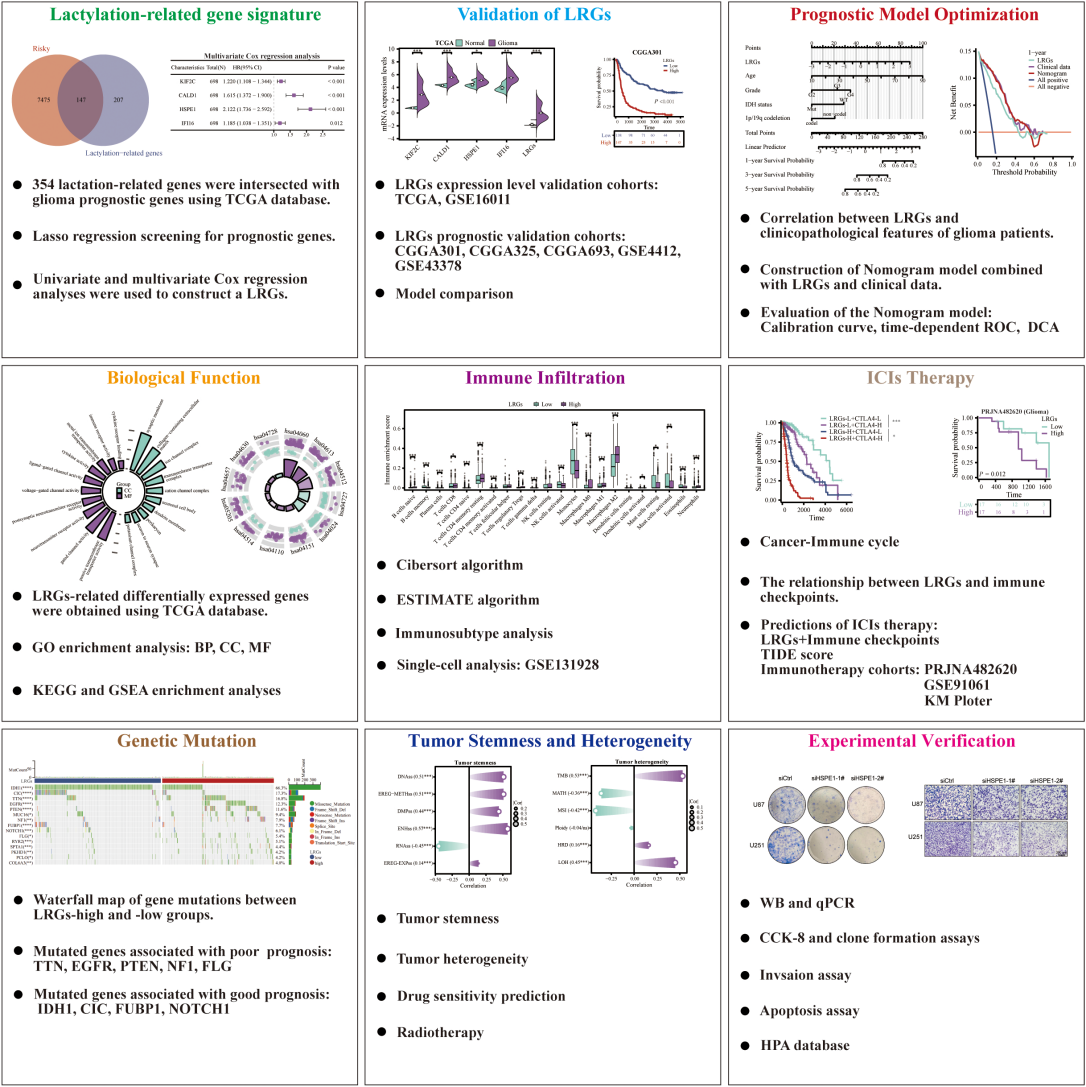
Flow chart of this study.

**Figure 2:**
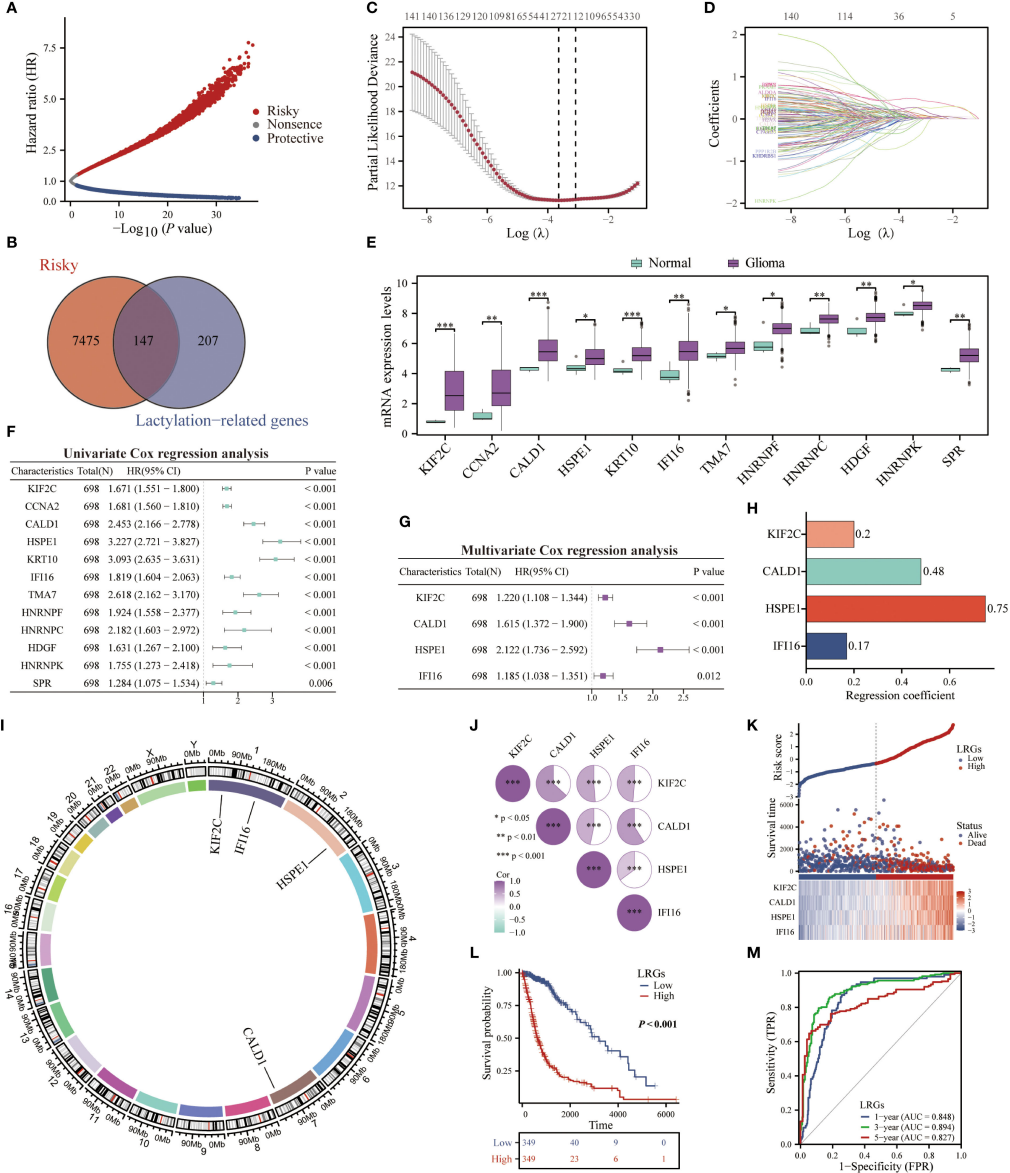
Construction of a LRGs signature based on the TCGA glioma cohort. **(A)** The volcano map displayed the genes that affected the survival of glioma patients. **(B)** The Venn diagram showing the intersection of risky genes affecting the survival of glioma patients with lactylation-related genes. **(C, D)** Lasso regression analysis further screened for prognosis genes among lactylation-related genes. **(E)** Comparison of differences in the expression of lactylation-related genes between normal brain and glioma tissues. **(F, G)** Univariate and multivariate Cox regression analyses were performed to construct a LRGs signature in glioma. **(H)** The band plot displayed the regression coefficients of each gene in the LRGs. **(I)** The chromosome map showing specific localization of KIF2C, CALD1, HSPE1, and IFI16. **(J)** The heatmap showing the correlation between these four genes. **(K)** The scatter plot demonstrating the difference in survival time and number of deaths of patients in the LRGs-high and -low groups. **(L)** Kaplan-Meier survival analysis was performed to demonstrate the difference in overall survival of glioma patients between the LRGs-high and -low groups using the TCGA cohort. **(M)** Time-dependent ROC demonstrating the accuracy of LRGs in predicting 1-, 3-, and 5-year survival in glioma patients. *p < 0.05, **p < 0.01, ***p < 0.001.

The workflow of this study was depicted in **Figure 1**. To establish a LRGs signature predictive of glioma prognosis, we employed the TCGA glioma cohort as the training set. Initial analysis identified 7,622 risk-associated genes and 5,264 protective genes (**Figure 2A**). Intersection of the 7,622 risk-associated genes with 354 known lactylation-related genes yielded 147 overlapping candidates (**Figure 2B**). Subsequent LASSO regression refined this set to 28 potential prognostic genes (**Figures 2C, D**). Differential expression analysis revealed significant upregulation of nearly half these genes in glioma versus normal brain tissues (**Figure 2E**). Through integrated univariate and multivariate Cox proportional hazards regression analyses, four lactylation-related genes—KIF2C, CALD1, HSPE1, and IFI16—emerged as core biomarkers, forming the basis of the LRGs signature (**Figures 2F, G**). The specific Cox regression coefficients were applied in the formula: LRGs score = (0.20 × KIF2C expression) + (0.48 × CALD1 expression) + (0.75 × HSPE1 expression) + (0.17 × IFI16 expression) – 8 (constant) (**Figures 2H**). These four genes localized to distinct chromosomal loci: KIF2C (1p34), CALD1 (7q33), HSPE1 (2q33), and IFI16 (1q23) (**Figure 2I**). Heatmap analysis demonstrated significant positive co-expression correlations among all four genes within glioma tissues (**Figure 2J**). Scatterplot illustrated that high-LRGs score patients experienced both elevated mortality rates and reduced survival durations (**Figure 2K**). Consistent with this, Kaplan-Meier survival curve revealed markedly inferior overall survival for patients stratified into the LRGs-high group compared to the -low group (**Figure 2L**). The LRGs signature exhibited robust predictive capacity, with time-dependent ROC analysis yielding area under the curve (AUC) values of 0.848 (95%CI: 0.809 - 0.887), 0.894 (95%CI: 0.860 - 0.928), and 0.827 (95%CI: 0.775 - 0.880) for 1-, 3-, and 5-year survival, respectively (**Figure 2M**).

### Validation of the LRGs signature

**Figure 3:**
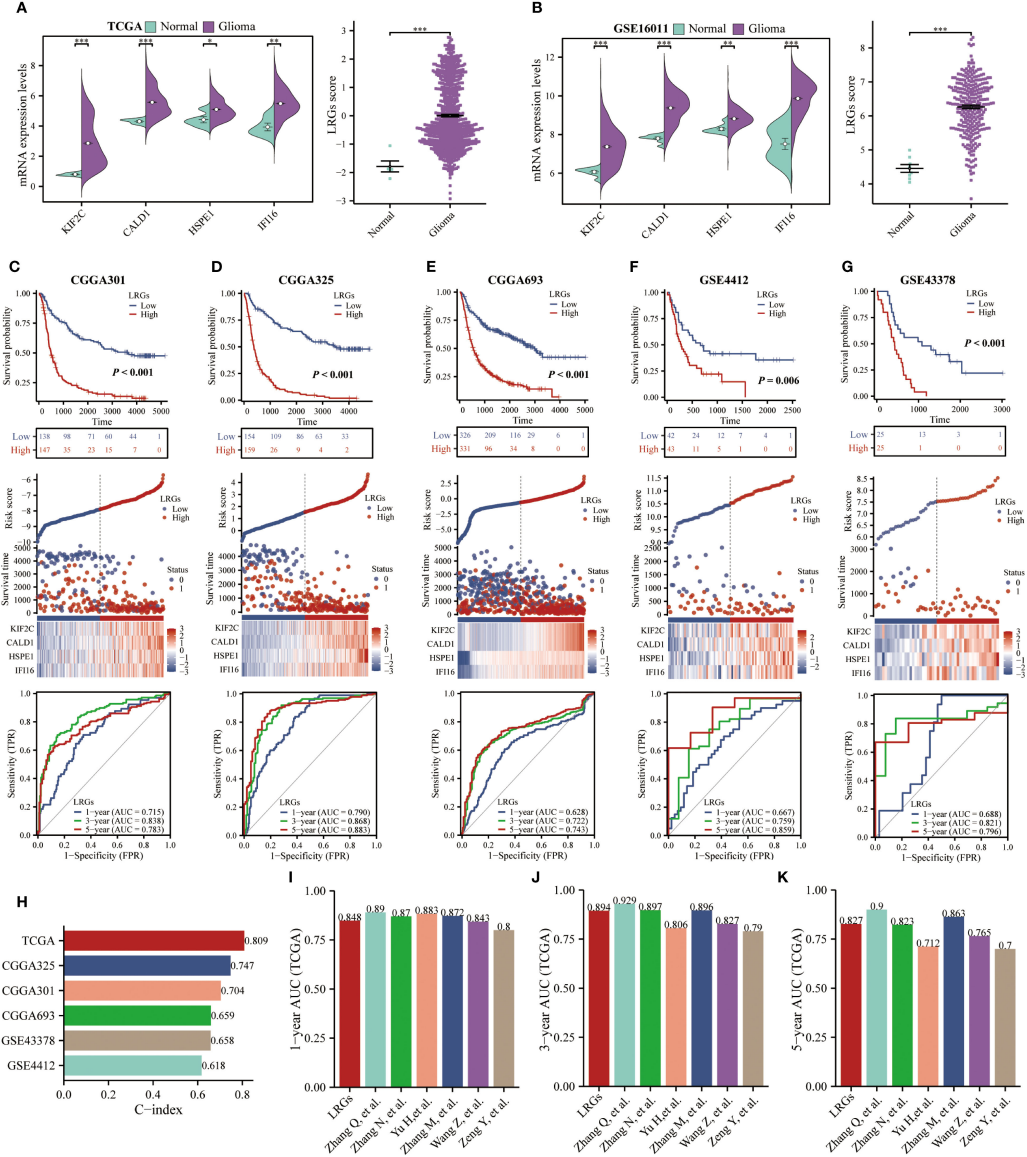
Validation of the LRGs expression levels and prognostic value was based on multiple cohorts. **(A, B)** TCGA and GSE16011 cohorts were used to validate the expression levels of KIF2C, CALD1, HSPE1, IFI16, and the LRGs score between normal brain and glioma tissues, respectively. **(C–G)** The scatter plots, Kaplan-Meier, and time-dependent ROC curves were utilized to validate the prognostic value of the LRGs in glioma using the CGGA301, CGGA325, CGGA693, GSE4412, and GSE43378 cohorts. **(H)** C-index of the LRGs model in five glioma cohorts. **(I–K)** The predictive accuracy of our generated LRGs in predicting 1-, 3-, and 5-year survival in glioma patients was compared to models constructed by others. *p < 0.05, **p < 0.01, ***p < 0.001.

To validate the prognostic robustness of the LRGs signature, we first quantitatively evaluated expression patterns in TCGA and GSE16011 cohorts. This analysis confirmed significantly upregulation of LRGs score and its core genes (KIF2C, CALD1, HSPE1, and IFI16) in glioma versus normal tissues (**Figures 3A, B**). We subsequently validated the LRGs prognostic value in five independent glioma cohorts, including CGGA301, CGGA325, CGGA693, GSE4412, and GSE43378. Patients stratified into LRGs-high group exhibited consistently inferior overall survival and elevated mortality incidence compared to -low group. Time-dependent ROC analyses demonstrated sustained predictive accuracy, with AUC values exceeding 0.700 for 3- and 5-year survival probabilities in all validation cohorts (**Figures 3C–G**). Further quantification via Harrell’s concordance index yielded C-index values of 0.809 (95%CI: 0.797 - 0.822; TCGA), 0.747 (95%CI: 0.731 - 0.762; CGGA325), 0.704 (95%CI: 0.686 - 0.722; CGGA301), 0.659 (95%CI: 0.644 - 0.673; CGGA693), 0.658 (95%CI: 0.620 - 0.695; GSE43378), and 0.618 (95%CI: 0.581 - 0.655; GSE4412), affirming the model’s discriminative capacity (**Figure 3H**). Comparative analysis against established prognostic signatures revealed that our LRGs model achieved comparable prognostic efficacy with greater parsimony, outperforming multi-gene constructs including those by Zhang Q et al. (16 genes) (19), Zhang N et al. (5 gene pairs) (20), and Zhang M et al. (10 genes) (21) in feature economy (**Figures 3I–K**).

### Prognostic model refinement through multivariate integration

**Figure 4:**
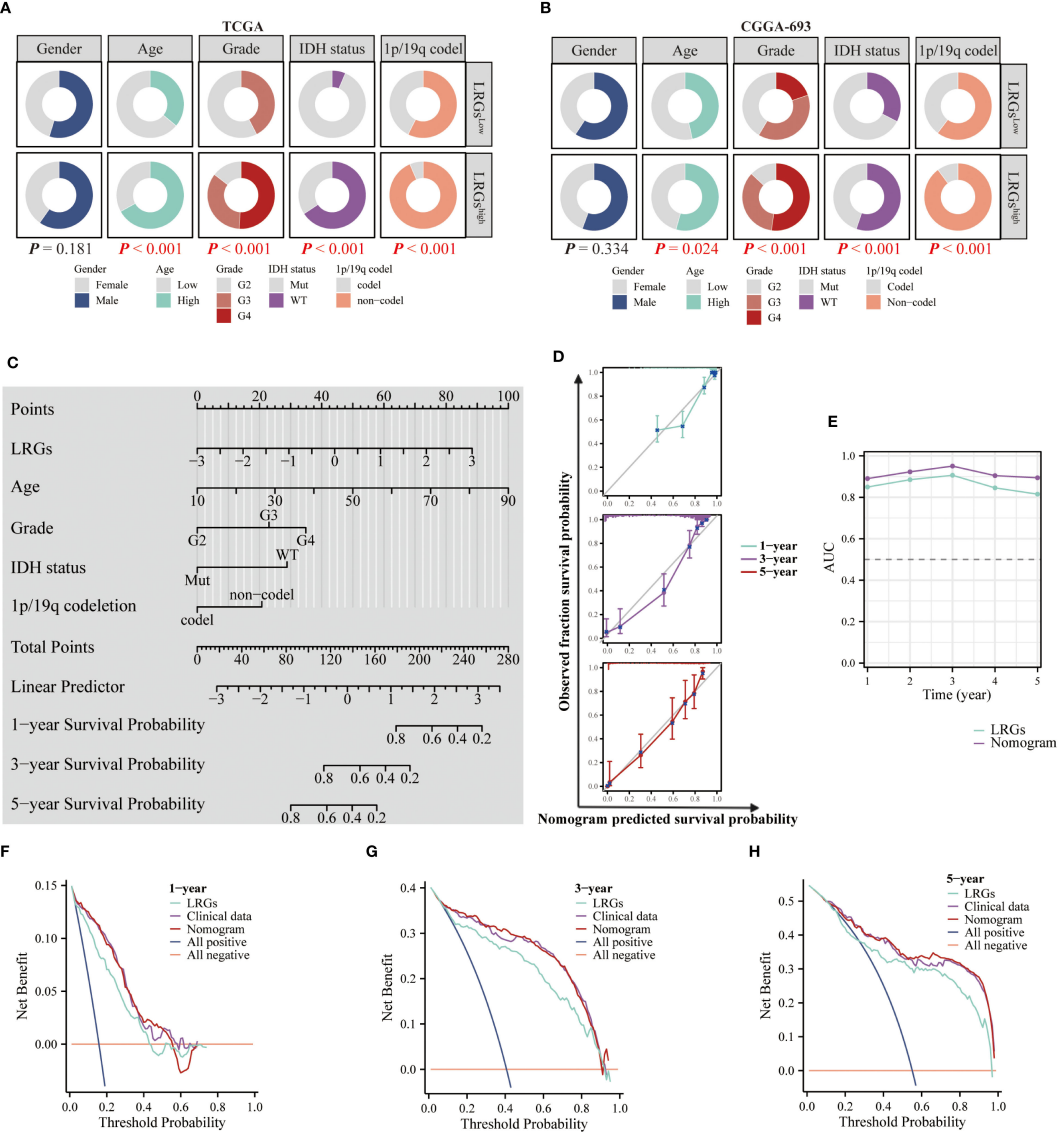
Clinical relevance of the LRGs and optimization of the nomogram. **(A, B)** Differences in clinicopathologic characteristics of glioma patients between the LRGs-high and -low groups using the TCGA and CGGA693 cohorts, including gender, age, WHO grade, IDH status, and 1p/19q co-deletion. **(C)** Five variables, LRGs score, gender, age, WHO grade, IDH status, and 1p/19q co-deletion were used to construct a nomogram model based on the TCGA glioma cohort. **(D)** Calibration curves. **(E)** Time-dependent AUC curves. **(F–H)** DCA curves for 1-, 3- and 5-year, respectively.

To optimize the precision of our LRGs signature, we systematically evaluated clinicopathological covariates influencing glioma outcomes. Comparative analysis of TCGA and CGGA693 cohorts revealed significant differences in multiple clinical characteristics between LRGs-high and -low glioma patient groups—including age, WHO grade, IDH status, and 1p/19q co-deletion—but showed no association with gender distribution (**Figures 4A, B**). Next, univariate and multivariate Cox regression analyses confirmed LRGs score, age, WHO grade, IDH status, and 1p/19q codeletion as independent risk factors (**Table 2**). These variables were subsequently integrated into a comprehensive nomogram, with calibration curves demonstrating excellent agreement between predicted and observed survival probabilities (**Figures 4C, D**). The composite model achieved a Harrell’s concordance index (C-index) of 0.860 (95% CI: 0.850 - 0.871). Time-dependent ROC analysis further validated its predictive efficacy, with AUC values exceeding 0.800 for 1- to 5-year survival predictions (**Figure 4E**). DCA demonstrated that the nomogram provided superior clinical net benefit compared to using the LRGs signature alone across most threshold probabilities, particularly at 3- and 5-year time points, outperforming both all-treat and all-none reference strategies (**Figures 4F–H**). Collectively, the integrated nomogram demonstrated enhanced prognostic capability and clinical applicability for glioma risk stratification.

**Table 2:** Univariate and multivariate Cox regression analyses were used to screen for risk factors for glioma using the TCGA glioma cohort.

Characteristics	Total (N)	Univariate analysis		Multivariate analysis	
		Hazard ratio (95% CI)	*P* value	Hazard ratio (95% CI)	*P* value
LRGs score	695	2.710 (2.411 - 3.047)	< 0.001	1.456 (1.192 - 1.780)	< 0.001
Gender	695				
Male	398	Reference			
Female	297	0.793 (0.621 - 1.012)	0.062		
Age	695	1.066 (1.056 - 1.076)	< 0.001	1.032 (1.020 - 1.045)	< 0.001
Grade	634				
G2	223	Reference		Reference	
G3	243	2.999 (2.007 - 4.480)	< 0.001	1.801 (1.162 - 2.791)	0.009
G4	168	18.615 (12.460 - 27.812)	< 0.001	2.435 (1.357 - 4.371)	0.003
IDH status	685				
Mut	439	Reference		Reference	
WT	246	8.551 (6.558 - 11.150)	< 0.001	2.089 (1.339 - 3.259)	0.001
1p/19q codel	688				
Codel	170	Reference		Reference	
Non-codel	518	4.428 (2.885 - 6.799)	< 0.001	1.699 (1.010 - 2.857)	0.046

### Functional enrichment implicates the LRGs signature in immune pathway regulation

**Figure 5:**
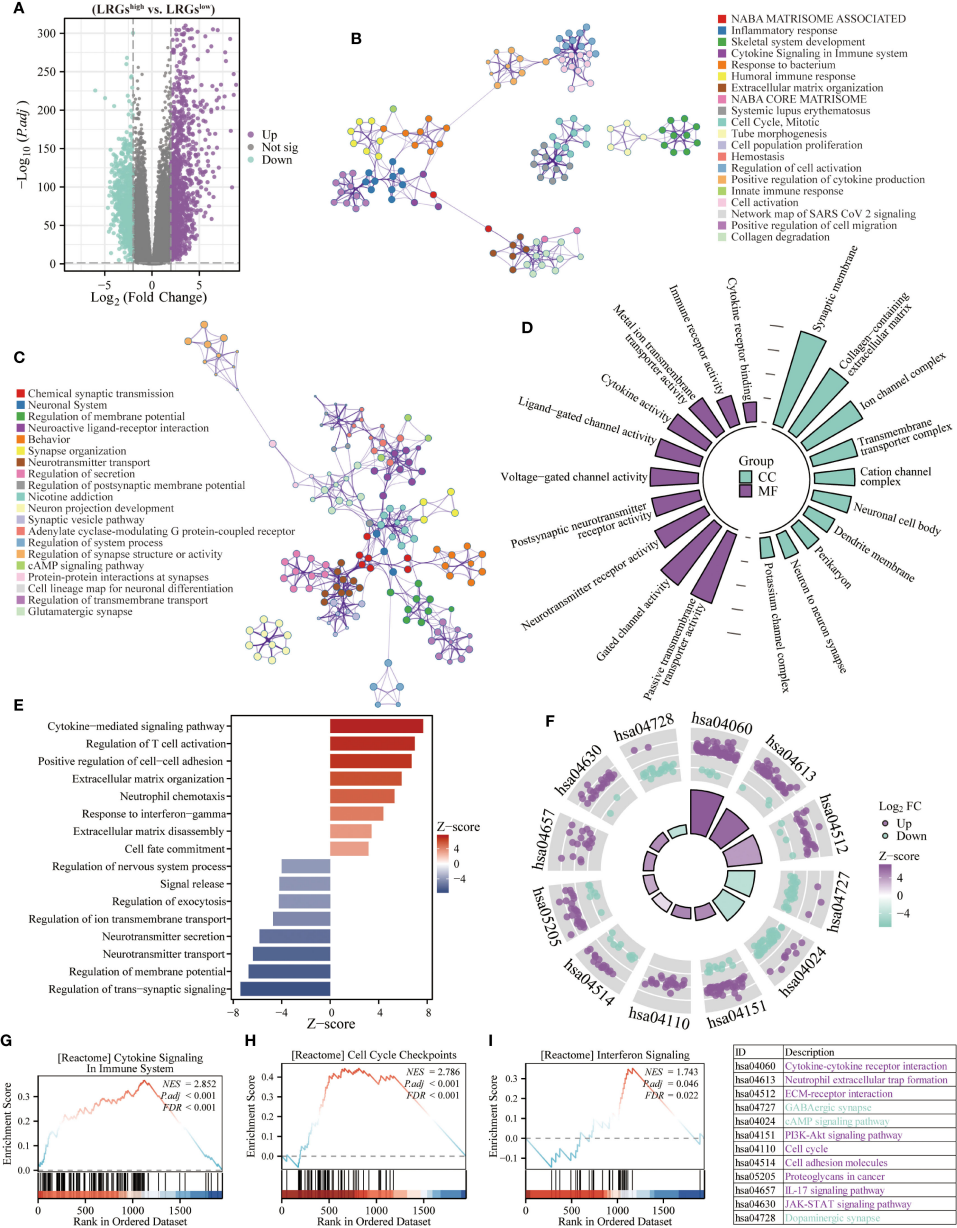
Biological functions of the LRGs. **(A)** The volcano map showing differentially expressed genes between LRGs-high and -low groups using the TCGA glioma cohort. **(B, C)** The Metascape platform was used for enrichment analysis of upregulated and downregulated genes, respectively. **(D)** The radiographic histogram demonstrating LRGs-related genes were subjected to GO enrichment analysis, including MF and CC. **(E)** The Z-score plot demonstrating LRGs-related genes were performed for GO enrichment analysis, including BP. **(F)** The chordal plot showing KEGG analysis of LRGs-related genes. **(G–I)** GSEA analysis of LRGs-related genes.

Given the significant prognostic association of the LRGs signature, which prompting further investigation into its functional underpinnings in glioma biology. Differential expression analysis between LRGs-high and -low groups identified 1,169 significantly upregulated and 803 downregulated genes (**Figure 5A**). Notably, these upregulated genes demonstrated significant enrichment in immune signaling pathways, including inflammatory response, cytokine signaling, and immune response functions, while downregulated genes were primarily implicated in synaptic function and neurotransmitter transmission (**Figures 5B, C**). GO analysis revealed distinct compartmentalization characteristics, with cellular components primarily localized to synaptic membranes, collagen-containing extracellular matrices, and transmembrane transporter complexes (**Figure 5D**). Molecular functions predominantly involved passive transmembrane transporter activity, gated channel function, and cytokine binding (**Figure 5D**). Biological process annotation demonstrated positive regulation of cytokine-mediated signaling, T cell activation, and interferon-γ response pathways, while negatively regulating trans-synaptic signaling, membrane potential modulation, and neurotransmitter transport (**Figure 5E**). KEGG pathway analysis further confirmed involvement in oncogenic and immune signaling cascades, including PI3K-AKT, IL-17, and JAK-STAT pathways (**Figure 5F**). GSEA of hallmark gene sets verified significant associations with cytokine signaling, cell cycle checkpoint regulation, and interferon response pathways (**Figures 5G–I**).

### The LRGs signature associates with immunosuppressive microenvironment

**Figure 6:**
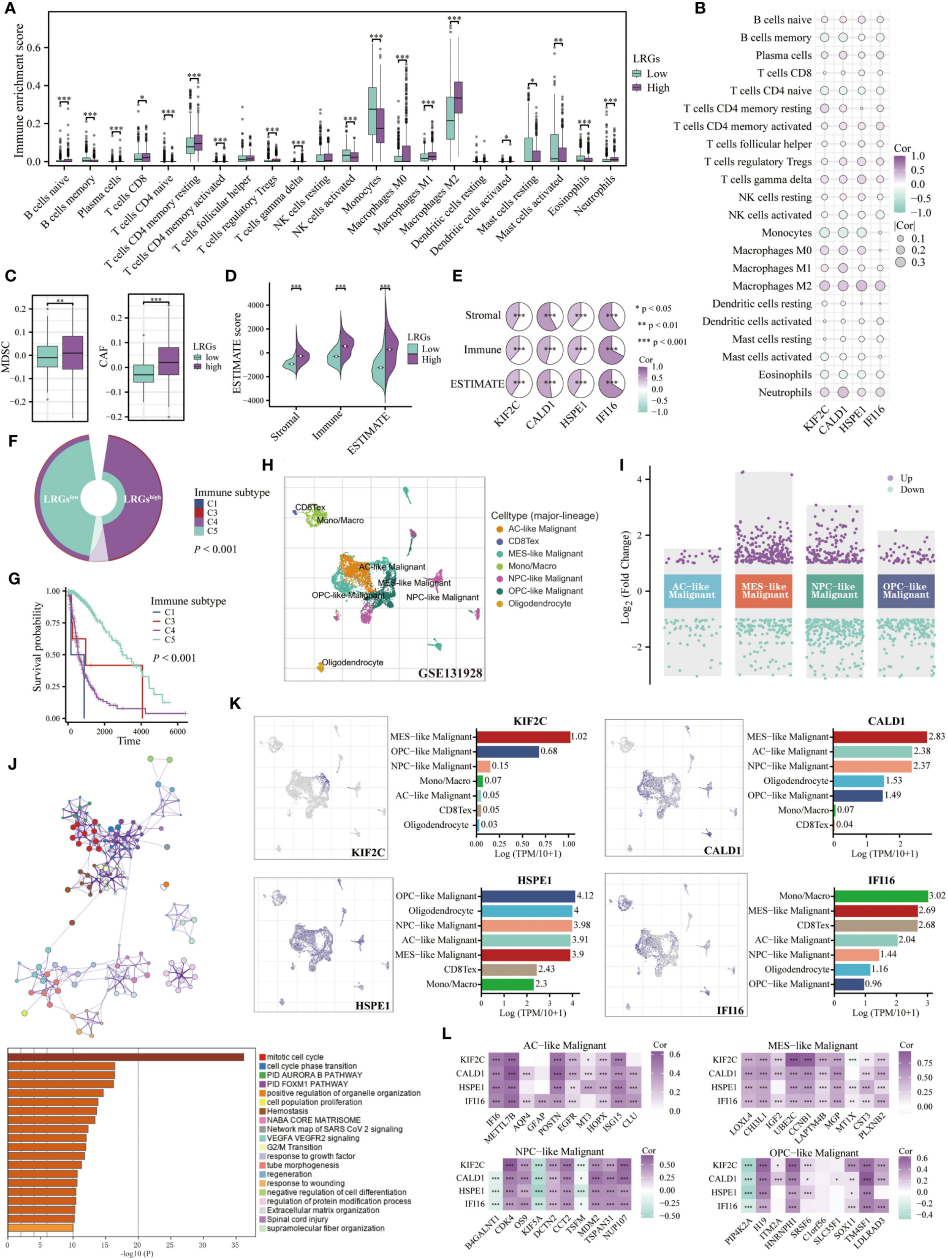
Role of the LRGs in the tumor microenvironment. **(A)** Differences in infiltration of immune cells between LRGs-high and -low groups based on the Cibersort algorithm. **(B)** The heatmap showing the correlation of the LRGs core genes with immune cells. **(C, D)** Differences in MDSCs, CAFs, and ESTIMATE scores between LRGs-high and -low groups. **(E)** The heatmap showing the correlation of the LRGs core genes with ESTIMATE scores. **(F)** Differences in immune subtypes between LRGs-high and -low groups. **(G)** Kaplan-Meier curve showing the effect of immune subtypes on overall survival of glioma patients. C1: wound healing, C2: IFN-gamma dominant, C3: inflammatory, C4: lymphocyte depleted, C6: TGF-β dominant. **(H, I)** The volcano plot displayed differentially expressed genes across various types of malignant cells based on the GSE131928 single-cell cohort. **(J)** Enrichment analysis of upregulated genes in malignant cells based on the Metascape platform. **(K)** The expression abundance of KIF2C, CALD1, HSPE1, and IFI16 across various cell types. **(L)** KIF2C, CALD1, HSPE1, and IFI16 exhibited correlations with the top 10 expressed genes in AC-like, MES-like, NPC-like, and OPC-like malignant cells, respectively. *p < 0.05, **p < 0.01, ***p < 0.001.

Given the established connection between the LRGs and immune pathways, we systematically profiled tumor-infiltrating immune cells using the Cibersort algorithm. Patients in the LRGs-high group exhibited significant enrichment of immunosuppressive populations, including M0, M1, and M2 macrophages, neutrophils, regulatory T cells (Tregs), γδ T cells, and resting memory CD4^+^ T cells. In contrast, other patients showed preferential infiltration of monocytes, activated NK cells, and activated mast cells (**Figure 6A**). The expression of LRGs core genes demonstrated significant positive correlations with pro-tumoral macrophages (particularly M2-polarized subtypes), neutrophils, Tregs, and γδ T cells, while being inversely correlated with monocytes, memory B cells, and naïve CD4^+^ T cells (**Figure 6B**). Complementary analyses using the TIDE and ESTIMATE algorithms further revealed increased infiltration of MDSCs and CAFs, along with elevated stromal, immune, and ESTIMATE scores in glioma with high LRGs score (**Figures 6C, D**). Each of these ESTIMATE metrics showed positive correlations with the expression of all four LRGs core genes (**Figures 6E**). Immunophenotypic stratification analysis revealed that patients in the LRGs-low group predominantly exhibited the immunologically favorable C5 subtype, while those in the -high group were significantly enriched for the poor-prognosis C4 subtype (**Figures 6F, G**).

To elucidate the cellular expression patterns of LRGs core genes, we utilized the TISCH2 platform to analyze the GSE131928 cohort, with a focus on genes specifically expressed in malignant cell populations (**Figure 6H**). The results revealed 27 significantly upregulated and 55 downregulated genes in AC-like malignant cells; 246 upregulated and 147 downregulated genes in MES-like malignant cells; 176 upregulated and 269 downregulated genes in NPC-like malignant cells, and 61 upregulated and 172 downregulated genes in OPC-like malignant cells (**Figure 6I**). We further extracted these upregulated genes for enrichment analysis and found that they were primarily involved in regulating cell cycle and oncogenic pathways, such as the FOXM1 and VEGFA pathways (**Figure 6J**). The LRGs core genes were specifically expressed in various malignant cell populations, with HSPE1 showing particularly prominent expression. Additionally, IFI16 was also expressed in monocyte/macrophage lineages and exhausted T cell compartments (**Figure 6K**). Based on these findings, we extracted the top 10 upregulated genes expressed in each malignant cell subtype and observed significant positive correlations with the four LRGs core genes, except for B4GALNT1, KIF5A, and TSFM in NPC-like malignant cells and PIP4K2A in OPC-like malignant cells (**Figure 6L**).

### The LRGs signature predicts response to immunotherapy

**Figure 7:**
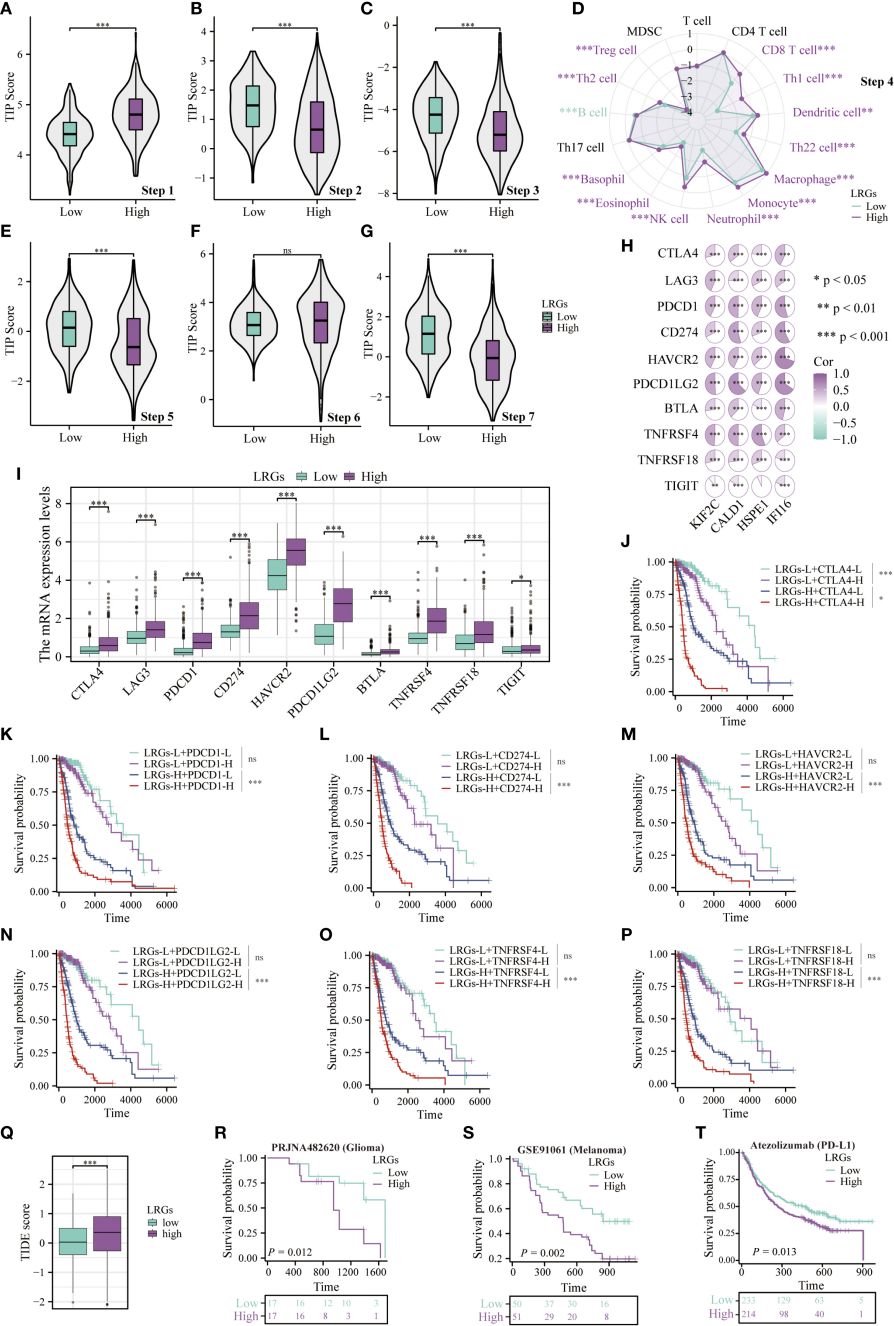
The LRGs predicted response to immunotherapy. Differences in cancer-immunity cycle between patients in the LRGs-high and -low groups were based on the TIP platform. **(A)** Step1: release of cancer cell antigens. **(B)** Step2: cancer antigen presentation. **(C)** Step3: priming and activation. **(D)** Step 4: trafficking of immune cells to tumors. **(E)** Step 5: infiltration of immune cells into tumors. **(F)** Step 6: recognition of cancer cells by T cells. **(G)** Step7: killing of cancer cells. **(H)** The heatmap demonstrating the correlation of KIF2C, CALD1, HSPE1, and IFI16 with multiple immunosuppressive checkpoints. **(I)** Differential expression of immunosuppressive checkpoints in LRGs-high and -low groups. **(J–P)** Kaplan-Meier curves demonstrating the LRGs combined with CTLA4, PDCD1, CD274, HAVCR2, PDCD1LG2, TNFRSF4, or TNFRSF18 respectively, to predict overall survival in glioma patients. **(Q)** Differences in TIDE scores between LRGs-high and -low groups. **(R–T)** Differences in survival between patients in the LRGs-high and -low groups receiving immunotherapy were analyzed based on the glioma (PRJNA482620), melanoma (GSE91061), and atezolizumab pan-cancer (KM Plotter) cohorts. *p < 0.05, **p < 0.01, ***p < 0.001.

To evaluate the role of the LRGs in tumor immunity, we characterized cancer-immunity cycle dysregulation between LRGs-high and -low groups. Despite elevated tumor antigen release, patients in the LRGs-high group exhibited significantly impaired antigen presentation and processing capacity (**Figures 7A–C**). Concurrently, these patients demonstrated heightened immune cell recruitment to tumor peripheries but diminished intratumoral infiltration (**Figures 7D, E**). Although T-cell recognition of tumor cells remained comparable between the two groups, patients in the LRGs-high group exhibited significantly reduced cytotoxic efficacy against cancer cells (**Figures 7F, G**). Subsequent analysis revealed significant positive correlations between the LRGs core genes and multiple immune checkpoints, with these checkpoints demonstrating marked upregulation in the LRGs-high group (**Figures 7H, I**). Kaplan-Meier analysis demonstrated poorer survival outcomes in patients with concurrently elevated expression of both the LRGs and immune checkpoints, including CTLA-4, PDCD1, CD274, HAVCR2, PDCD1LG2, TNFRSF4, or TNFRSF18 (**Figures 7J–P**). Multi-platform validation incorporating TIDE algorithmic assessment, glioma, melanoma, and atezolizumab pan-cancer immunotherapy cohorts consistently showed that patients in the LRGs-high group exhibited elevated TIDE score, shortened survival duration, and attenuated therapeutic responsiveness to immune checkpoint blockade (**Figures 7Q-T**). These findings collectively established the LRGs signature as a biomarker for response to immunotherapy in glioma.

### Genetic variation of the LRGs

**Figure 8:**
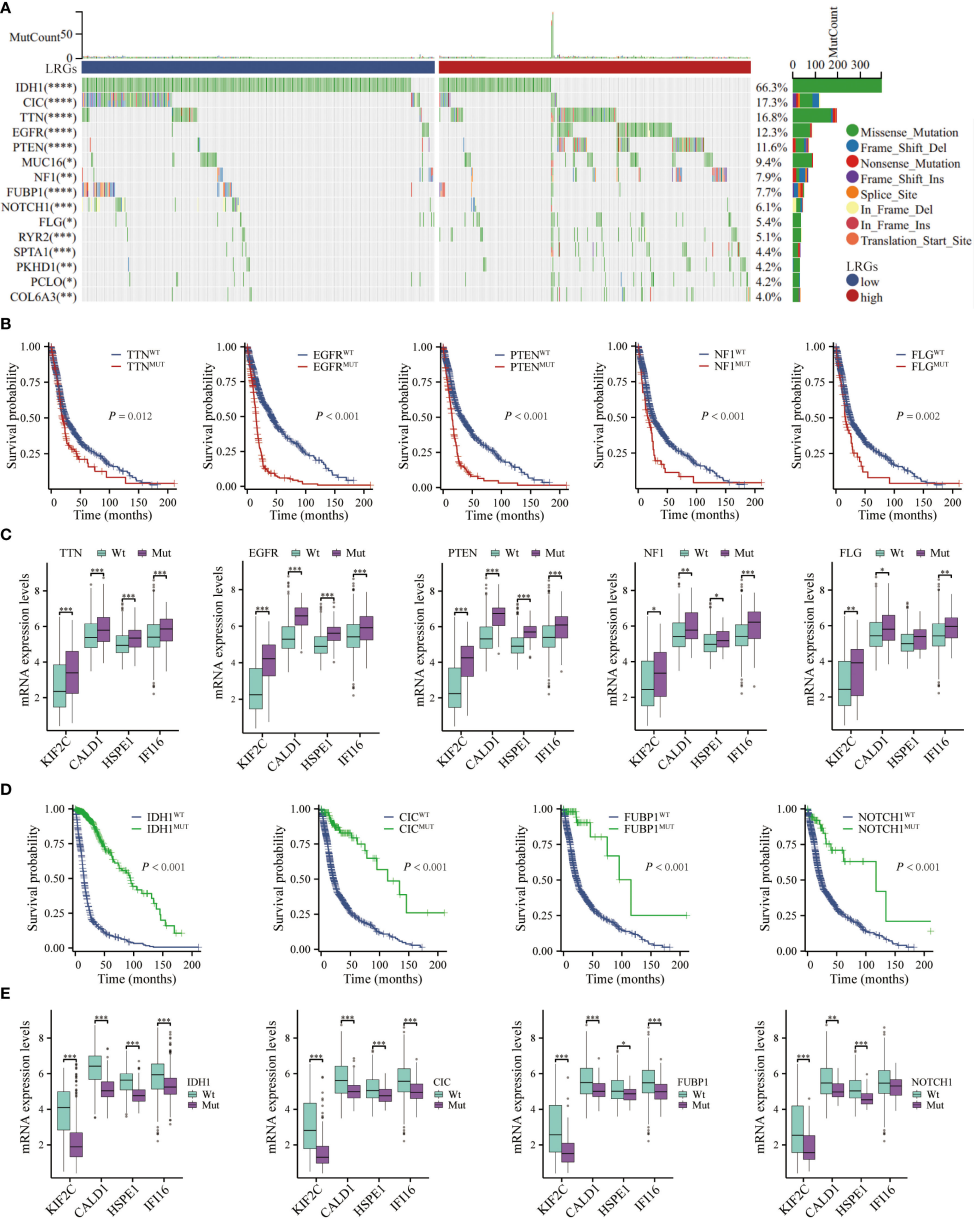
Genetic mutation analysis. **(A)** The waterfall depicted genetic mutations differences in glioma patients between LRGs-high and -low groups. **(B)** Kaplan-Meier curves demonstrating the difference in overall survival of glioma patients between the TTN, EGFR, PTEN, NF1, and FLG mutant- and wild-type groups. **(C)** Differential expression of KIF2C, CALD1, HSPE1, and IFI16 between TTN, EGFR, PTEN, NF1, and FLG mutant- and wild-type groups, respectively. **(D)** Kaplan-Meier curves demonstrating the difference in overall survival of glioma patients between the IDH1, CIC, FUBP1, and NOTCH1 mutant- and wild-type groups. **(E)** Differential expression of KIF2C, CALD1, HSPE1, and IFI16 between IDH1, CIC, FUBP1, and NOTCH1 mutant- and wild-type groups, respectively. *p < 0.05, **p < 0.01, ***p < 0.001.

Given the established link between genetic alterations and oncogenesis, we examined LRGs-associated genomic variations in glioma progression using comprehensive mutational profiling (**Figure 8A**). Mutations in critical driver genes, such as TTN, EGFR, PTEN, NF1, and FLG, demonstrated significant association with adverse overall survival outcomes (**Figure 8B**). The expression levels of LRGs core genes were significantly elevated in most oncogenic mutation groups, with the exception of HSPE1 suppression in FLG-mut group (**Figure 8C**). Conversely, mutations in IDH1, CIC, FUBP1, or NOTCH1 conferred favorable prognoses, correlating with downregulation of these LRGs-associated markers, though IFI16 expression remained unaltered in NOTCH1-mut group (**Figures 8D, E**). These findings position LRGs as molecular integrators of genetic variation influencing glioma pathogenesis.

### Associations between the LRGs and tumor stemness, genomic heterogeneity, and therapeutic sensitivity

**Figure 9:**
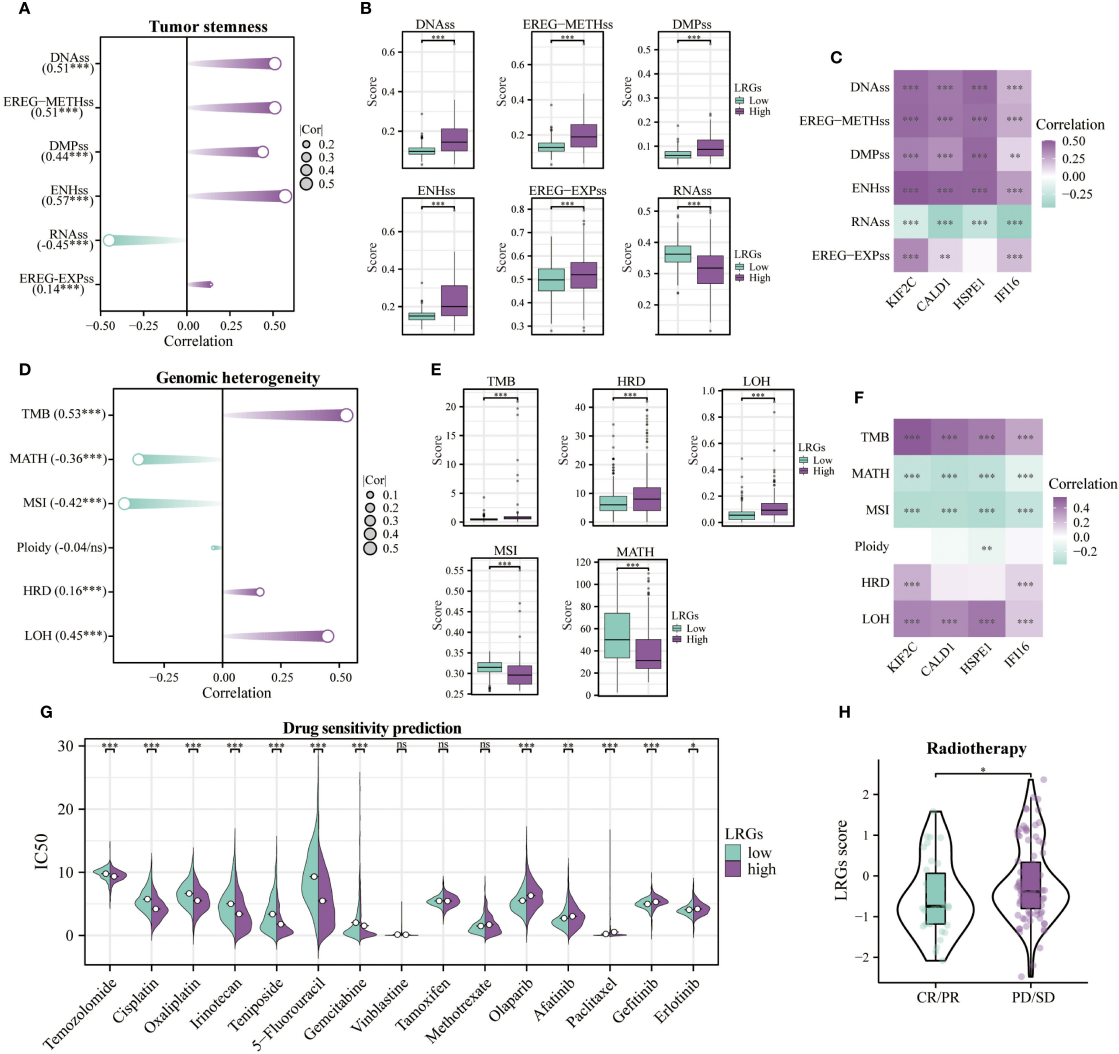
Tumor stemness, genomic heterogeneity, and drug sensitivity analyses. **(A)** Correlation of the LRGs with tumor stemness-related indicators, including DNAss, EREG-METHss, DMPss, ENHss, RNAss, and EREG-EXPss. **(B)** Differences in tumor stemness-related indicators between LRGs-high and -low groups. **(C)** Correlation analysis between the LRGs core genes and tumor stemness-related indicators. **(D)** Correlation of the LRGs with genomic heterogeneity-related indicators, including TMB, MATH, MSI, Ploidy, HRD, and LOH. **(E)** Differences in genomic heterogeneity-related indicators between LRGs-high and -low groups. **(F)** Correlation analysis between the LRGs core genes and genomic heterogeneity-related indicators. **(G)** Differences in sensitivity to common chemotherapeutic agents between LRGs-high and -low groups. **(H)** Differences in LRGs score between PD/SD and PR/CR groups of glioma patients treated with radiotherapy. *p < 0.05, **p < 0.01, ***p < 0.001.

Patients with elevated tumor stemness scores tend to have poorer prognosis, and these scores can predict tumor metastasis and recurrence. Comprehensive analysis revealed that the LRGs and its core genes showed positive correlations with DNAss, EREG-METHss, DMPss, ENHss, and EREG-EXPss, but negative correlation with RNAss, suggesting that the LRGs may promote tumor cell dedifferentiation and enhance stemness (**Figures 9A–C**). Tumor patients exhibiting high heterogeneity levels face increased risks of recurrence and mortality. Results of heterogeneity analysis revealed that the LRGs and their core genes were positively correlated with TMB, HRD, and LOH, while showing negative correlations with MATH and MSI, suggesting that tumors with high LRGs score exhibited greater genomic instability, higher mutational burden, and potential homologous recombination deficiency (**Figures 9D–F**). Chemosensitivity analysis indicated that patients in the LRGs-high group exhibited enhanced sensitivity to temozolomide, cisplatin, oxaliplatin, irinotecan, teniposide, 5-fluorouracil, and gemcitabine, but developed resistance to olaparib, afatinib, paclitaxel, gefitinib, and erlotinib (**Figure 9G**). Notably, the LRGs score was significantly elevated in the radiotherapy non-responder (PD/SD) group compared to the responder (PR/CR) group, further establishing LRGs as biomarkers of therapeutic resistance **Figure 9H**.

### HSPE1 promotes tumorigenesis in glioma

**Figure 10:**
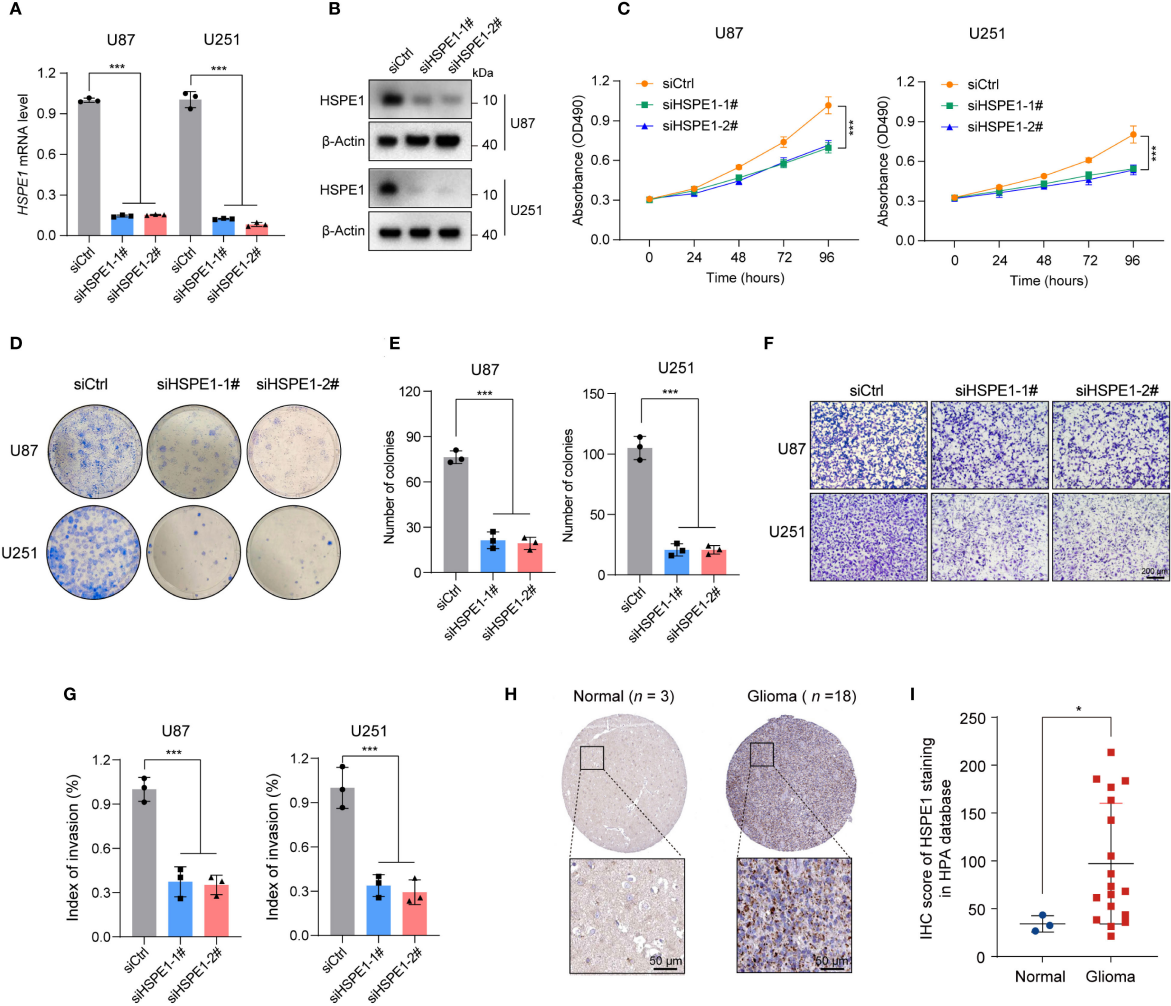
Knockdown of HSPE1 expression inhibited proliferation and invasion of glioma U251 and U87 cells *in vitro*. **(A)** HSPE1 mRNA levels in U87 and U251 cells after knockdown. **(B)** Western Blot showing HSPE1 protein levels in U87 and U251 cells after knockdown. **(C)** CCK-8 assay for cell proliferation in U87 and U251 after HSPE1 knockdown. **(D)** Colony formation assay showing the number of colonies in U87 and U251 after HSPE1 knockdown. **(E)** Quantification of colonies formed in U87 and U251 cells after HSPE1 knockdown. **(F)** Transwell invasion assay showing cell migration in U87 and U251 cells. **(G)** Quantification of cell migration in U87 and U251 cells. **(H)** Representative immunohistochemistry staining of HSPE1 in glioma and normal tissue in HPA database. **(I)** IHC score of HSPE1 staining in the HAP database from glioma and normal tissues. Data in A, C, E, G, and I are presented as the mean ± SD. *p < 0.05, ***p < 0.001. One-way ANOVA with Tukey’s test for A, C, E, and G (n = 3). Two-tailed student’s t-test for I.

Given that HSPE1, a core LRGs gene, demonstrated the highest hazard ratio in multivariate Cox analysis, exhibited high expression abundance in malignant cells, and has an undefined functional role in gliomagenesis, we selected HSPE1 for further investigation into its ability to affect glioma cells. In U87 and U251 glioma cell models, we successfully knocked down HSPE1 expression, as verified by qPCR and Western blot analysis (**Figures 10A, B**). Functional characterization demonstrated that HSPE1 ablation significantly attenuated malignant phenotypes, suppressing cellular proliferation in CCK-8 assay (**Figure 10C**), reducing clonogenic capacity in colony formation assay (**Figures 10D, E**), and markedly attenuating invasive potential in transwell assay (**Figures 10F, G**). However, HSPE1 knockdown showed no significant effect on the apoptosis of glioma cells (**Supplementary Figure S1**). Complementing these deficits observed in *in vitro* functional assays, clinical validation utilizing the Human Protein Atlas (HPA) database revealed significant overexpression of HSPE1 in glioma specimens compared to normal brain tissue, which was further confirmed by immunohistochemical (IHC) analysis of tissue microarrays (**Figures 10H, I**). In summary, these integrated mechanistic and clinical findings establish HSPE1 as a critical mediator of gliomagenesis within the lactylation-related oncogenic network.

## Discussion

Lactylation, a post-translational modification, has emerged as a key regulator of glioma biology, particularly during metabolic reprogramming, thereby controlling tumor growth and metastasis. For example, lactate promoted the synthesis of nucleoside triphosphates essential for the proliferation of H3K27M-mutant glioma cells through lactylation-mediated activation of nucleoside diphosphate kinase NME1 (22). Lactylation of c-Myc maintained the stability of c-Myc, which promoted the migration and invasion of GBM cells (23). Additionally, lactylation modification maintains the stemness of glioma cells and drug resistance. For instance, PTBP1 lactylation was found to facilitate glioma stem cell maintenance through PFKFB4-driven glycolysis (24). Glycolytic reprogramming-induced XRCC1 lactylation induced therapeutic resistance in ALDH1A3-overexpressing glioblastoma (12). Histone H3K9 lactylation was shown to confer temozolomide resistance in glioblastoma through LUC7L2-mediated retention of the MLH1 intron (25). Briefly, lactylation modification plays a critical role in the development of glioma, and an in-depth exploration of the relationship between lactylation and glioma can help unravel the biological mechanisms underlying tumorigenesis.

In this study, we focused on investigating the expression patterns of Lactylation-related genes and constructed a LRGs signature based on four genes—KIF2C, CALD1, HSPE1, and IFI16—that were highly expressed in glioma tissues, which effectively stratified patient risk and assisted clinicians in developing personalized treatment strategies. Notably, our model demonstrated advantages in both predictive accuracy and model simplicity when compared side-by-side with other established prognostic models for glioma, enhancing its clinical applicability. In contrast to the lactylation-related glioma prognostic model developed by researchers such as Wu Z et al. (14), our approach incorporated a larger number of lactylation-related genes, providing superior predictive performance for patient prognosis and immunotherapy response. Furthermore, we demonstrated that HSPE1 knockdown significantly inhibited the proliferation and invasion of glioma cells. This multi-dimensional analysis offered a more comprehensive perspective on glioma biology and held potential for enabling more individualized treatment strategies.

One of the most notable advancements in our model was the incorporation of the nomogram, which integrated the LRGs score with critical clinicopathological factors such as age, WHO grade, IDH status, and 1p/19q codeletion. This integration significantly improved the predictive accuracy of the model, making it more practical for clinical use. Our nomogram, with a C-index of 0.860, demonstrated strong predictive power, particularly in predicting 1- to 5-year survival, with AUC values exceeding 0.8. These impressive metrics underscored the model’s ability to provide precise prognosis predictions that are essential for individualized patient management. Additionally, the time-dependent ROC and DCA further demonstrated that the nomogram significantly outperformed both the LRGs score alone and traditional clinical models in predicting survival at the 3- and 5-year time points, thus establishing its value as a robust tool for guiding treatment decisions and improving patient outcomes.

Studies have shown that the glioma microenvironment is immunosuppressive in nature, for example, regulatory T (Treg) cells prevented tumor cells from being directly killed by cytotoxic T lymphocyte (CTL) cells by depleting CTL-mediated immunosuppression in the glioma microenvironment (26). Importantly, lactylation modification played a key role in the glioma microenvironment, for example, lactate induced epigenetic reprogramming of glioma cells through histone lactylation, which led to transcriptional upregulation of CD47 to inhibit phagocytosis and promote immune evasion (27). Additionally, LDHA-mediated lactate production impaired tumor immune surveillance by T cells and NK cells (28). In the present study, we revealed that the LRGs core genes were associated with various immunosuppressive cells, such as M2 macrophages, MDSCs, and CAFs. It has been reported that hypoxia-mediated lactate accumulation was taken up by macrophages, which subsequently induces M2 macrophage polarization by regulating TNFSF9 expression and promoted the secretion of cytokines such as IL-10, TGF-β, and VEGF, thereby facilitating malignant progression of glioma (29, 30). These findings suggested that LRGs core genes may play a critical role in maintaining the immunosuppressive properties of the glioma microenvironment.

Recently, with the groundbreaking discovery of lymphatic vessels in the central nervous system, the emergence of immunotherapy has undoubtedly brought new hope for patients with refractory glioma. For instance, therapeutic inhibition of PD-L1 or CTLA-4 significantly reduced the number of tumor-infiltrating Treg cells and improved long-term survival in mouse glioma models (31). Lactylation-related genes, such as SLC16A3, TRIM28, and BRD4, can promote immune evasion by increasing PD-L1 expression abundance or diminishing the efficacy of *anti*-PD-1 therapy (32–34). Furthermore, lactylation modification had been investigated in immunotherapy, for example, CCR8 lactylation can impair the effectiveness of CAR-T therapy against glioblastoma (35). Notably, this study revealed that the LRGs core genes were significantly associated with immunosuppressive checkpoints, and glioma patients in the LRGs-high exhibited lower response rates and overall remission rates to immunotherapy. These findings suggested that the LRGs may play a role in tumor immune evasion, tumorigenesis, and resistance to immunotherapy.

One of the key strengths of our study was the experimental validation of the critical gene within the LRGs. Specifically, we provided functional evidence that HSPE1 played an essential role in glioma progression. HSPE1, a co-chaperonin involved in mitochondrial protein import and macromolecular assembly, worked together with Hsp60 to facilitate the correct folding of imported proteins. HSPE1 had been found to be aberrantly expressed in various cancers and was associated with poor prognosis (36). Notably, its expression was significantly elevated in multiple GBM cell lines (37). However, the functional role of HSPE1 in glioma has remained unclear until now. Our findings established that HSPE1 was a key player in glioma progression, and its upregulation contributed to tumor growth and invasiveness. This experimental validation strengthened the translational potential of our findings and provided a direct link between computational predictions and experimental biology, highlighting HSPE1 as a promising therapeutic target for glioma treatment.

By integrating machine learning, genomic data, tumor microenvironment features, and experimental validation, our study offered a comprehensive, multi-faceted approach to glioma prognosis. This refined LRGs model not only improved prediction accuracy but also enhanced our ability to understand the underlying biological mechanisms, ultimately facilitating the development of more effective, personalized therapies for glioma patients. Further validation through prospective clinical trials will be crucial for translating this model into routine clinical practice.

## Limitation

Our study has some limitations. First, the analysis is based on retrospective data, and prospective clinical validation is needed to confirm the value of the lactylation-based prognostic model in real-world settings. Additionally, while the model demonstrated strong predictive ability, further studies are required to identify additional lactylation-related genes that could improve its accuracy. The functional validation of HSPE1, particularly *in vivo*, is another critical step to further solidify the biological relevance of these markers. Moreover, the specific mechanisms through which lactylation modification regulates immune microenvironment components, such as M2 macrophage polarization, require further in-depth investigation.

## Conclusion

In summary, we developed a prognostic risk model for glioma based on the four lactylation-related genes, integrating clinical features, tumor microenvironment, genomic heterogeneity, and drug sensitivity. The model exhibited strong predictive accuracy, providing a comprehensive approach for personalized treatment. Experimental validation of HSPE1 revealed its crucial role in glioma progression, supporting its potential as a therapeutic target. Our findings highlight the value of combining molecular signatures and machine learning to improve the prognosis of glioma and guide clinical decision-making.

## Data Availability

The data presented in the study are deposited in the Figshare repository. Accession Link: https://doi.org/10.6084/m9.figshare.30104440.v1/
