# Supplementary figures and images for: Deciphering the lactylation landscape in glioma: a novel gene signature predicts patient survival and immunotherapy sensitivity
Source: Front Immunol. 2025 Sep 16;16:1664347. doi: 10.3389/fimmu.2025.1664347 (PMC12479444; doi:10.3389/fimmu.2025.1664347)

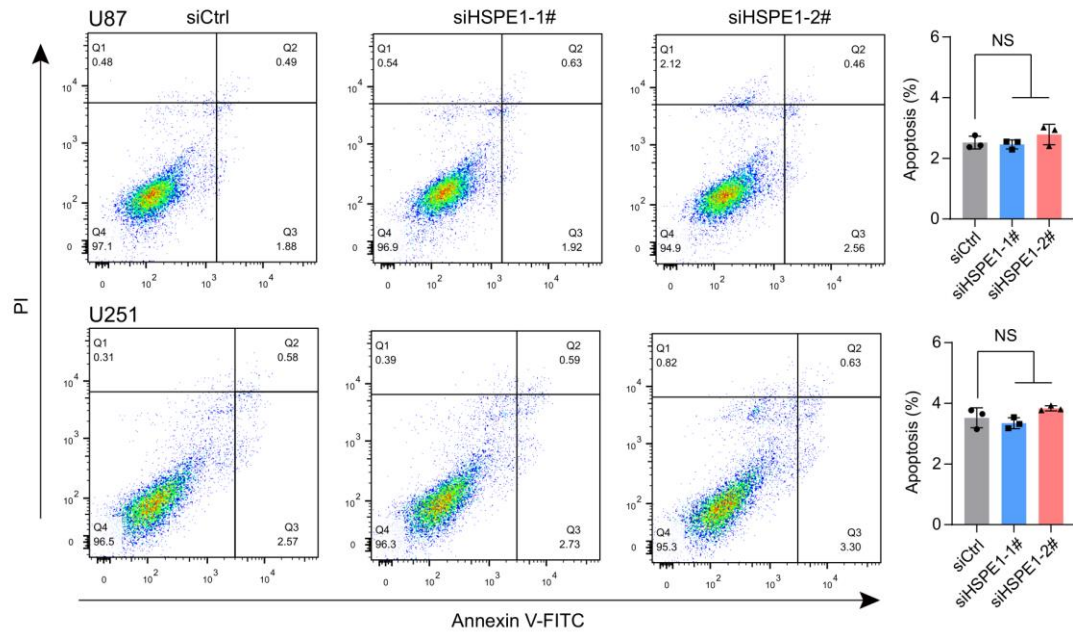

Figure S1. The effect of HSPE1 knockdown on apoptosis in U87 and U251 glioma cells. NS, not significant.

Supplement: Supplementary file 1 [file DataSheet1.pdf]
